# Efficacy of Intravenous Ketamine in Status Epilepticus: A Systematic Review and Meta-Analysis With Pediatric Subgroup Analysis

**DOI:** 10.7759/cureus.99264

**Published:** 2025-12-15

**Authors:** Bader F Aldhafeeri, Omar Alkhaldi, Faisal Alajmi, Lolwah Alenezi, Shumoukh Alajmi, Esra Alenezi, Shouq Almotawtah, Abdullah Mohammed Alenezi, Zahraa Alnakkas, Jassim Alqallaf, Fajer Altammar

**Affiliations:** 1 Department of Pediatrics, New Jahra Hospital, Al Jahra, KWT; 2 Department of Pediatrics, King Saud Medical City, Riyadh, SAU; 3 Department of Pediatrics, Farwaniya Hospital, Al Farwaniyah, KWT; 4 Pediatric Intensive Care Unit, New Jahra Hospital, Al Jahra, KWT

**Keywords:** ketamine infusion, pediatric seizure, refractory status epilepticus, status epilepticus, super-refractory status epilepticus

## Abstract

Status epilepticus (SE), particularly its refractory (RSE) and super-refractory (SRSE) forms, remains a critical neurological emergency with high morbidity and mortality, and treatment options become limited when conventional GABAergic anesthetics lose effectiveness or cause hemodynamic instability. Ketamine, through its antagonism of N-methyl-D-aspartate receptors, offers a mechanistically distinct and potentially advantageous therapeutic option. This systematic review and meta-analysis evaluated the efficacy of intravenous ketamine for terminating seizures in SE across adult and pediatric populations, synthesizing data from 10 studies involving 388 patients. A random-effects model demonstrated an overall seizure termination rate of 56% (95% CI: 51-61) with no heterogeneity. Subgroup analysis showed efficacy rates of 59% in adults and 50% in children, with no statistically significant difference between age groups, although adults showed a numerically higher response. Despite wide variability in dosing regimens and frequent use of concomitant anesthetics, seizure control was consistently observed across cohorts. Most included studies were of good methodological quality. These findings support intravenous ketamine as a clinically meaningful and hemodynamically favorable treatment option for RSE and SRSE when standard therapies fail and highlight the need for prospective, standardized, and especially pediatric-focused research to optimize dosing, timing, and long-term outcome assessment.

## Introduction and background

Status epilepticus (SE) is a serious medical neurological emergency characterized by prolonged or persistent seizures without regaining consciousness, associated with substantial morbidity and mortality rates [1]. According to the International League Against Epilepsy, SE is defined by the failure of normal seizure-terminating mechanisms, with operational time points of ≥5 minutes for generalized convulsive SE and longer durations for other types. A significant number of patients progress to refractory status epilepticus (RSE), defined as seizures that continue despite adequate doses of a benzodiazepine and one appropriate antiseizure medication, or to super-refractory status epilepticus (SRSE), where seizures continue despite the administration of first- and second-line treatments and following the standardized treatment protocols [2,3]. The management of these severe forms of SE presents a clinical challenge, frequently requiring patients to be managed in the intensive care unit with continuous intravenous anesthetic drugs.

The management of RSE and SRSE commonly relies on traditional anti-epileptic drugs such as midazolam and barbiturates, and they remain the mainstay treatment in such conditions [4]. These drugs work mainly by activating GABA receptors; however, their therapeutic efficacy can decrease over time due to receptor desensitization during prolonged seizures [5,6]. Alternative treatment options are needed due to severe systemic complications that are associated with prolonged infusions of these medications.

Ketamine, which blocks N-methyl-D-aspartate (NMDA) receptors, provides a mechanistically distinct therapeutic approach. Ketamine works by blocking excitotoxic glutamatergic signaling, which offers a theoretical benefit for patients who do not respond to GABA-based treatments [7]. Clinical evidence also suggests that ketamine may offer neuroprotective properties and relative hemodynamic stability compared to traditional anesthetic drugs. Consequently, these properties have led to its increasing use as salvage therapy in RSE and SRSE across both adult and pediatric populations [8].

Ketamine efficacy in terminating seizures remains variable, despite multiple observational studies and case series that have reported the use of intravenous ketamine in SE. Moreover, the overall effectiveness of ketamine remains uncertain due to differences in study design, dosing protocols, and patient characteristics. In particular, the role of ketamine in the management of SE in children remains unclear, and understanding how children respond to it compared to adults, due to age-related differences in how the drugs work and the underlying causes of seizures, is essential.

Although previous reviews have summarized existing evidence, significant gaps remain. Smaller datasets, fewer contemporary studies, and minimal exploration of differences between pediatric and adult patients limited earlier reviews. Importantly, no prior systematic review has comprehensively analyzed the comparative efficacy of ketamine across age groups using an updated evidence base. Pediatric patients represent a particularly understudied population, and age-related pharmacodynamic and etiological differences may influence treatment response.

We performed a systematic review and meta-analysis to evaluate the efficacy of intravenous ketamine for the management of SE, with a specific focus on comparing outcomes between children and adult patients.

## Review

Methods

Data Collection and Search Strategy

A comprehensive literature search was conducted in PubMed, Embase, Scopus, Web of Science, and the Cochrane Library, including studies up to June 30, 2025. The search strategy combined both controlled vocabulary (MeSH terms) and free-text keywords related to status epilepticus and ketamine. The primary MeSH terms included “Status Epilepticus” and “Ketamine”. Search strategy: (("Status Epilepticus") OR ("status epilepticus" OR "refractory status epilepticus" OR "super-refractory status epilepticus" OR “RSE” OR “SRSE” OR “Seizures”)) AND (("Ketamine") OR (ketamine OR "Anesthetics, Dissociative"[Mesh] OR esketamine OR "NMDA antagonist")). We included only studies published in English. This review was conducted in accordance with the PRISMA 2020 guidelines. The review protocol was not registered in PROSPERO.

Eligibility Criteria

Studies were considered eligible if they met the following criteria: (1) included adult and/or pediatric patients diagnosed with SE, including RSE and SRSE forms; (2) investigated intravenous ketamine as the intervention for seizure termination; (3) reported efficacy outcomes defined as seizure termination or control after ketamine administration; and (4) were designed as randomized controlled trials, cohort studies, case-control studies, or case series with at least ten patients. We restricted inclusion to studies published in English, with no limitations on publication date. Studies were excluded if they (1) were case reports, narrative reviews, or systematic reviews; (2) were conference abstracts lacking sufficient data; or (3) involved animal or preclinical models.

Study Selection

All identified records were imported into the Rayyan website for deduplication. Two reviewers independently screened titles and abstracts for eligibility, followed by a full-text review of potentially relevant studies. Any disagreements were resolved by discussion or consultation with a third reviewer.

Data Extraction

Two reviewers independently extracted data from all included studies using a standardized Excel form. Extracted information included study characteristics (author, year, country, design, and setting), patient demographics (sample size, mean or median age, sex distribution, and adult versus pediatric subgroup), intervention details (ketamine dosing regimen, route, and infusion duration, as well as concomitant antiseizure therapies), and efficacy outcomes, defined as the number of patients achieving seizure termination according to the criteria used by each study. Any discrepancies were resolved through discussion or consultation with a third reviewer.

Risk of Bias

The methodological quality of the included observational studies was assessed using the NIH Quality Assessment Tool for Case Series and Cohort Studies. Each study was independently rated as good, fair, or poor quality by two reviewers, and discrepancies were resolved by consensus.

Statistical Analysis

We performed a single-arm meta-analysis to estimate the pooled proportion of seizure termination with intravenous ketamine. Proportions were stabilized using the Freeman-Tukey double arcsine transformation and pooled under a random-effects model (DerSimonian-Laird method) to account for between-study variability. Statistical heterogeneity was assessed using the I² statistic and Cochran’s Q test, with I² values of 25%, 50%, and 75% representing low, moderate, and high heterogeneity, respectively. Subgroup analysis was conducted by age group (adults vs. pediatrics). Potential small-study effects and publication bias were assessed using funnel plots and Egger’s regression test when ≥10 studies were available. All analyses were performed using R software (meta and metafor packages), and a two-tailed p-value <0.05 was considered statistically significant.

A single-arm meta-analysis model was selected because the included studies were predominantly observational and lacked consistent comparator groups, precluding the use of traditional comparative meta-analytic approaches. The studies demonstrated substantial clinical heterogeneity in patient characteristics, seizure etiologies, timing of ketamine initiation, dosing strategies, and concomitant antiseizure therapies. Given this variability, a single-arm model provided the most methodologically appropriate framework to synthesize the overall proportion of patients achieving seizure cessation. Meta-regression and comparative analyses were not feasible due to inconsistent reporting of covariates and the absence of uniform control arms across studies.”

Results

Search Results

**Figure 1:**
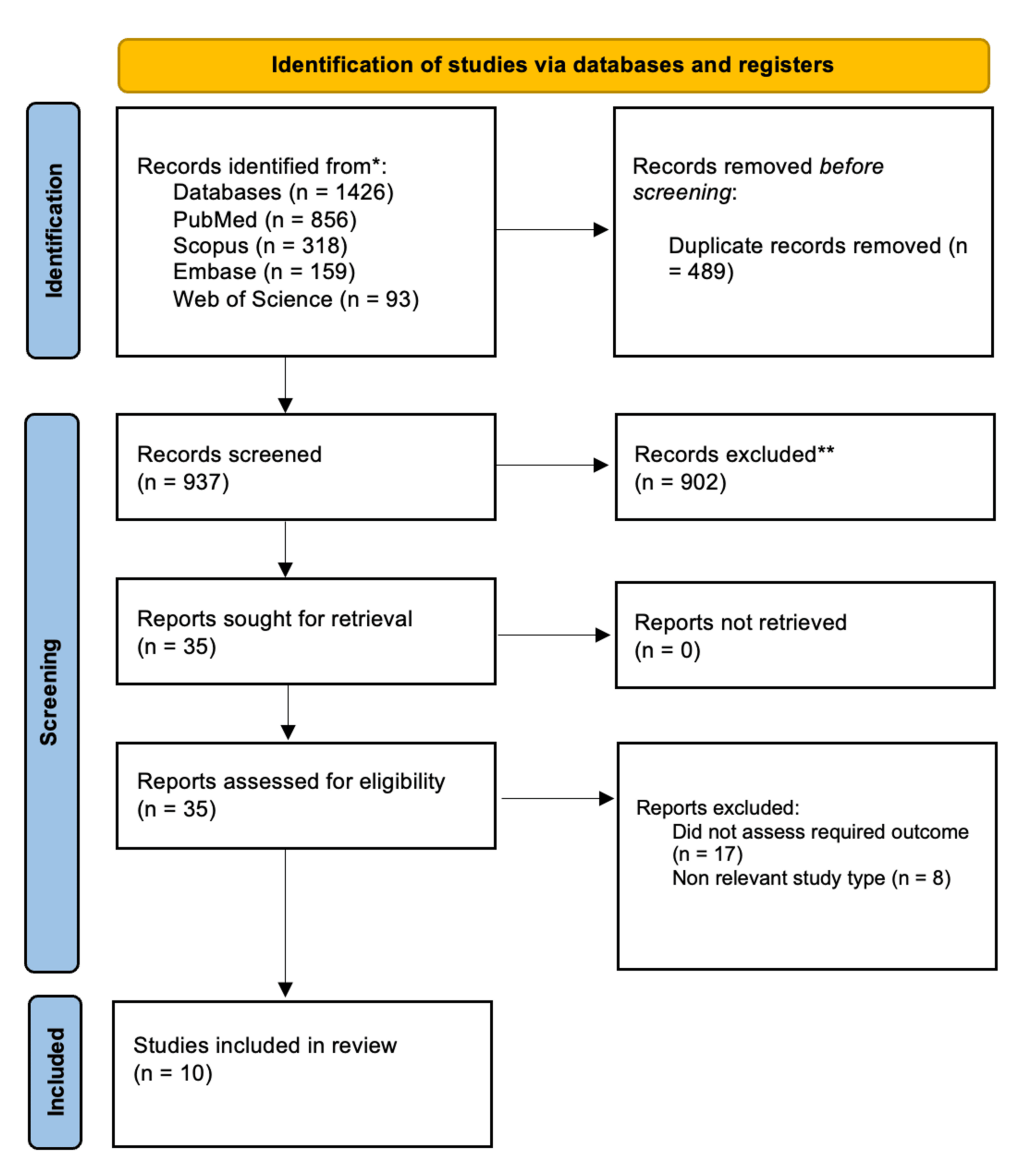
Flowchart of the selection process WOS: Web of Science

The systematic search identified 1,426 records across four electronic databases: PubMed (n = 856), Scopus (n = 318), Embase (n = 159), and Web of Science (n = 93). After removing 489 duplicate records, 937 unique titles and abstracts were screened; 902 were excluded as irrelevant, leaving 35 articles for full-text assessment. Following a detailed evaluation, 10 studies met the inclusion criteria and were included in the final analysis. The selection process is summarized in the PRISMA flow diagram (Figure 1).

Characteristics of Included Studies

Ten studies published between 2013 and 2024 were included [9-18], comprising 407 patients with RSE or SRSE treated with intravenous ketamine. Six studies evaluated adult populations (García-Ruiz et al., 2024; Höfler et al., 2016; Alkhachroum et al., 2020; Caranzano et al., 2022; Synowiec et al., 2013), while four included pediatric cohorts (Jacobwitz et al., 2022; Jacobwitz et al., 2024; Kuki et al., 2024; Ilvento et al., 2015; Gaspard et al., 2013).

Sample sizes ranged from 11 to 69 patients across single-center cohorts, with the largest study reporting 388 patients. Reported mean or median ages reflected wide variability, spanning from neonates (11 days old) to elderly adults (>70 years).

Ketamine regimens varied substantially. Bolus doses ranged from 0.5 to 2 mg/kg, while maintenance infusions ranged between 0.45 and 10 mg/kg/h, with some outliers up to 15 mg/kg/h. Duration of infusion varied from hours to over two weeks.

Seizure termination rates ranged from 39% to 64% in pediatric cohorts and from 50% to 64% in adult cohorts. Concomitant antiseizure medications and anesthetics (e.g., midazolam, propofol, pentobarbital, and thiopental) were almost universally co-administered, reflecting the refractory nature of the patient populations. The detailed baseline characteristics of all included studies are summarized in Table 1.

**Table 1:** Baseline characteristics SD: standard deviation, IQR: interquartile range, AEDs: anti-epileptic drugs, ICP: intracranial pressure, ASMs: anti-seizure medications, LEV: levetiracetam, VPA: valproic acid, LCM: lacosamide, CLZ: clozapine, PB: phenobarbital, SRSE: super-refractory status epilepticus, MMM: multimodality monitoring, RSE: refractory status epilepticus, RCSE: refractory convulsive status epilepticus

Study ID (author, year)	Study design	Sample size (total)	Age (mean ± SD or median (range)	Main ketamine dose used	Seizure termination	Concomitant therapies	Study aim	Study conclusion
García-Ruiz et al., 2024 [9]	Retrospective cohort	58	60.2 ± 15.7 years	Loading 1.34 ± 0.91 mg/kg; infusion mean 1.86 ± 1.19 mg/kg/h; max 2.74 ± 1.49 mg/kg/h	33 out of 58	Levetiracetam, lacosamide, valproate; anesthetics (midazolam, propofol) before ketamine	To evaluate the efficacy and safety of intravenous ketamine in patients with RSE and SRSE	Ketamine is an effective treatment with a good response rate and relatively few major adverse effects
Höfler et al., 2016 [10]	Retrospective cohort	42	Median 67 years (IQR 59.3–72.0)	Bolus 200 mg (n = 7); continuous infusion median 2.39 mg/kg/h (IQR 1.52–3.02), max median 2.55 mg/kg/h (IQR 2.09–3.22)	27 out of 42	Median 3 AEDs + 2 anesthetics prior	To explore the feasibility, efficacy, safety profile, and effect on outcome of intravenous (S)-ketamine in patients with RSE and SRSE	Intravenous (S)-ketamine had a good safety profile and achieved a 64% response rate in the treatment of RSE and SRSE. No adverse events were observed
Alkhachroum et al., 2020 [11]	Retrospective cohort	68	53 ± 19 years	Mean infusion 2.2 ± 1.8 mg/kg/h (range 0.2–10); median duration 2 days (IQR 1–4)	43 out of 68	All midazolam, plus propofol (36 pts), pentobarbital (10 pts); AEDs (levetiracetam, phenytoin, lacosamide, valproate)	To evaluate the efficacy of ketamine infusions in the treatment of SRSE and to assess the effects of high-dose ketamine on brain physiology, particularly using invasive MMM	Ketamine reduced seizure burden in SRSE and was hemodynamically safe, showing no increase in ICP or adverse cerebral effects
Caranzano et al., 2022 [12]	Prospective registry	11	48 ± 22 years	Median 5 mg/kg/h (range 2.5–15); duration median 2 days (range 1–16)	7 out of 11	All received anesthetics (propofol, midazolam) + multiple ASMs (LEV, VPA, LCM, CLZ, PB, etc.)	To evaluate the efficacy of ketamine in adult patients with SRSE using data from a prospective registry	Ketamine provided sustained seizure control in only a minority of SRSE cases, despite early and high-dose use. The findings suggest that prior studies may have overestimated ketamine's efficacy due to selection bias
Gaspard et al., 2013 [13]	Multicenter retrospective	60	Median 24 years	Loading median 1.5 mg/kg (max 5); Infusion median 2.75 mg/kg/h (range 0.05–10); duration 6h–27d	34 out of 60	Median 5 ASMs (range 1–11); anesthetics (propofol, midazolam, pentobarbital, thiopental)	To assess the patterns of use, efficacy, and safety of intravenous ketamine in the treatment of RSE across multiple centers	Continuous IV ketamine appeared relatively safe and moderately effective in controlling RSE, contributing to seizure control in 32% of cases
Synowiec et al., 2013 [14]	Retrospective cohort	11	Mean 52 ± 18 years	Bolus 1–2 mg/kg; infusion 0.45–2.1 mg/kg/h (mean 1.3); daily max 1392–4200 mg	11 out of 11	All on IV anesthetics before ketamine (propofol, lorazepam, midazolam, pentobarbital)	To retrospectively evaluate the use of intravenous ketamine as adjunctive therapy in patients with RSE, examining its efficacy, safety, and impact on hemodynamic stability over a 9-year period at a single institution	Ketamine successfully terminated RSE in all 11 patients, was well tolerated, and was not associated with any acute adverse events
Jacobwitz et al., 2022 [15]	Retrospective cohort	69	Median 0.7 years (neonates to children)	Infusion started at 1 mg/kg/h; max 1–7 mg/kg/h (median 2–3 mg/kg/h); boluses median 4 in first 24h	32 out of 69	Median 3 ASMs before ketamine; many also received midazolam, propofol, and pentobarbital, depending on sequence	To describe the impact of ketamine for RSE in children and neonates	Ketamine administration was associated with few adverse events, and seizures often terminated or improved after ketamine administration
Ilvento et al., 2015 [18]	Retrospective case series	19	Mean 5.3 years; range 2 months–11.5 years	Bolus 2–3 mg/kg ×2; infusion 5–10 mcg/kg/min, titrated up to 60; median 30 mcg/kg/min	14 out of 19	Midazolam, propofol, or thiopental often used	To evaluate the efficacy and safety of intravenous ketamine in treating RCSE in children	Ketamine was effective in resolving RCSE in 14 out of 19 episodes and allowed the avoidance of mechanical ventilation in selected patients
Jacobwitz et al., 2024 [16]	Retrospective cohort (comparative)	38	Median onset 292 days	Bolus 1 mg/kg; infusion start 1 mg/kg/h; median best response 2 mg/kg/h (IQR 1–3); max 6 mg/kg/h	23 out of 38	All failed ≥2 ASMs; median 3 (ketamine)	To compare the efficacy and adverse effects of ketamine vs midazolam as first-line anesthetic infusions in pediatric RSE	Ketamine was associated with higher seizure termination (61% vs 28%) and fewer adverse effects (3% vs 25%) than midazolam
Kuki et al., 2024 [17]	Retrospective cohort	18	Median 1y5m (11d–24y)	Bolus 0.5–2 mg/kg; infusion 1–6 mg/kg/h; median max 3.0 mg/kg/h (IQR 2.0–4.3); median duration 7.5 days	7 out of 18	Median 5 ASMs before ketamine; thiopental in 16 prior; 9 continued with ketamine; median 2 ASMs concomitant	To investigate the short-term benefits and adverse effects of ketamine in the treatment of pediatric and adolescent SRSE	Ketamine administration was associated with few serious adverse events and a significant reduction in seizure frequency in pediatric and adolescent patients with SRSE

Quality Assessment

Methodological quality was assessed using the NIH Quality Assessment Tool for Case Series and Cohort Studies. Of the 10 included studies, eight were rated good quality and two fair (Table 2).

**Table 2:** Quality assessment of the selected studies Each bar represents the proportion of responses for the 14 criteria: (N1) clear research question/objective; (N2) defined population; (N3) ≥50% participation; (N4) uniform recruitment criteria; (N5) sample size justification; (N6) exposure before outcome; (N7) adequate time frame; (N8) exposure levels analyzed; (N9) reliable exposure measures; (N10) repeated exposure assessment; (N11) reliable outcome measures; (N12) blinded outcome assessors; (N13) ≤20% loss to follow-up; and (N14) confounders measured/adjusted. Y: yes, N: no, CD: cannot determine References [9-18]

Study name	Q1	Q2	Q3	Q4	Q5	Q6	Q7	Q8	Q9	Q10	Q11	Q12	Q13	Q14	Overall quality
García-Ruiz et al., 2024	Y	Y	CD	Y	N	Y	Y	Y	Y	Y	Y	N	Y	Y	Good
Höfler et al., 2016	Y	Y	CD	Y	N	Y	Y	Y	Y	Y	Y	N	Y	Y	Good
Alkhachroum et al., 2020	Y	Y	CD	Y	N	Y	Y	Y	Y	Y	Y	N	Y	Y	Good
Caranzano et al., 2022	Y	Y	Y	Y	N	Y	Y	Y	Y	Y	Y	N	Y	Y	Good
Jacobwitz et al., 2022	Y	Y	CD	Y	N	Y	Y	Y	Y	Y	Y	N	Y	Y	Good
Gaspard et al., 2013	Y	Y	CD	Y	N	Y	Y	Y	Y	Y	Y	N	Y	Y	Good
Jacobwitz et al., 2024	Y	Y	CD	Y	N	Y	Y	Y	Y	Y	Y	N	Y	Y	Good
Kuki et al., 2024	Y	Y	Y	Y	N	Y	Y	Y	Y	Y	Y	N	Y	Y	Good
Synowiec et al., 2013	Y	Y	Y	Y	N	Y	Y	Y	Y	Y	Y	N	Y	N	Fair
Ilvento et al., 2015	Y	Y	Y	Y	N	Y	Y	Y	Y	Y	Y	N	Y	N	Fair

Meta-Analysis of Seizure Termination

**Figure 2:**
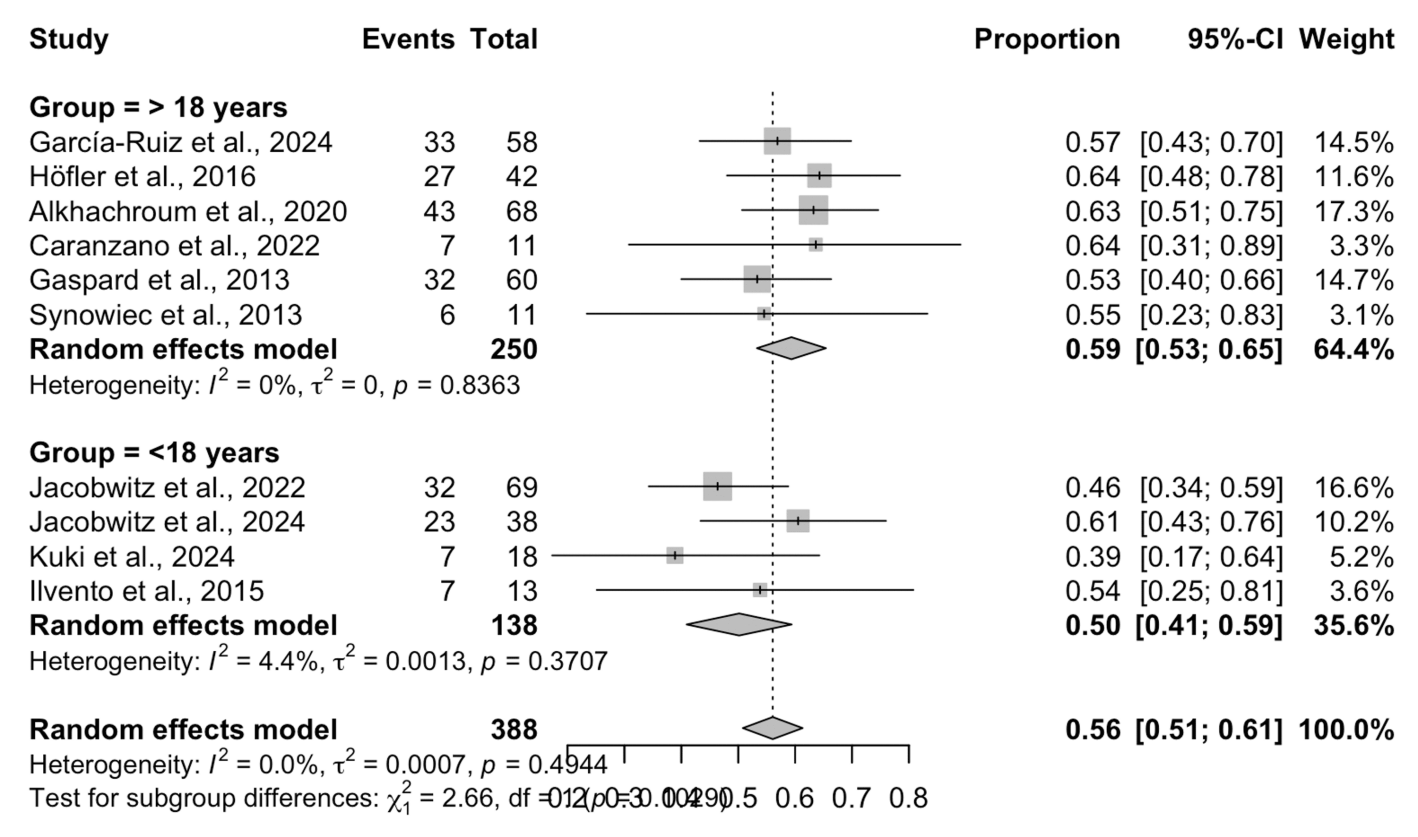
Forest plot of seizure termination after ketamine administration CI: confidence interval

The pooled random-effects analysis of the 10 included studies demonstrated an overall seizure termination rate of 0.56 (95% CI: 0.51-0.61), with no evidence of heterogeneity (I² = 0%, τ² = 0.0007, p = 0.4944). In the adult subgroup (>18 years; six studies, n = 250), the pooled efficacy rate was 0.59 (95% CI: 0.53-0.65), with consistent findings across individual studies (range: 0.53-0.64) and no heterogeneity (I² = 0%, p = 0.8363). In the pediatric subgroup (<18 years; four studies, n = 138), the pooled seizure termination rate was 0.50 (95% CI: 0.41-0.59), with slightly greater variability across studies (range: 0.39-0.61) but minimal heterogeneity (I² = 4.4%, p = 0.3707). The test for subgroup differences was not statistically significant (χ² = 2.66, df = 1, p = 0.1029), although there was a numerical trend toward higher efficacy in adults than in children (Figure 2).

Although the adult subgroup demonstrated a numerically higher seizure termination rate than the pediatric subgroup, the confidence intervals for both pooled estimates substantially overlapped, consistent with the non-significant subgroup comparison (p = 0.1029) and indicating no statistically reliable difference.

Discussion

RSE and SRSE are linked to substantial morbidity and mortality, posing a significant treatment challenge. When initial therapeutic measures fail, management becomes constrained, and the use of anesthetic agents is frequently complicated by hemodynamic compromise. Ketamine has been increasingly used in this setting owing to its unique NMDA receptor antagonism and favorable hemodynamic properties.

We integrated data from multiple cohorts that included both adult and pediatric patients in this systematic review and meta-analysis. Across studies, ketamine achieved clinically meaningful seizure termination, with reported response rates ranging from 7/11 patients (64%) in the prospective registry by Caranzano et al. (2022) [12] to 43/68 patients (63%) in the retrospective cohort of Alkhachroum et al. (2020) [11]. Similarly, García-Ruiz et al. (2024) [9] reported seizure control in 33/58 patients (57%), while Gaspard et al. (2013) [13] observed termination in 34/60 patients (57%) across a large multicenter cohort. Höfler et al. (2016) [10] demonstrated comparable results, with seizure cessation in 27/42 patients (64%). Pooled analysis of these studies confirmed the efficacy of intravenous ketamine as a treatment modality in both RSE and SRSE. Although the pooled estimates differed numerically between adults and pediatric patients, this difference was not statistically significant (p = 0.1029), and the substantial overlap in confidence intervals indicates that no definitive age-related difference in treatment response can be concluded.

Our results align with earlier observational studies and systematic reviews that reported seizure control rates of 30-60% with ketamine therapy [19]. The efficacy rates analyzed across our included studies exceeded the lower threshold of the reported range, further affirming ketamine’s therapeutic promise. Initial work by Gaspard et al. (2013) concentrated on the adult population; however, later series confirmed the therapeutic utility of ketamine in broader demographic groups, including pediatric patients, as evidenced by the work of Caranzano et al. (2022) and Garcia-Ruiz et al. (2024) [9,12,13]. When compared with anesthetic agents such as propofol, midazolam, or barbiturates, ketamine demonstrates noninferior efficacy and the additional benefit of a favorable hemodynamic profile [20]. This is especially important in critically ill patients, where hemodynamic instability is common. Compared to other antiepileptic drugs, such as anesthetic agents that may cause hypotension and worsen outcomes, ketamine offers a distinct advantage due to its favorable hemodynamic profile [21]. The mechanism of action underlying ketamine’s therapeutic efficacy is well-founded. During extended seizures, the efficacy of benzodiazepines is compromised by GABA receptor internalization, while excitatory neurotransmission through the NMDA receptors becomes progressively more prominent [22,23]. Ketamine, by directly blocking the NMDA receptors, counteracts this excitotoxic drive [24].

Ketamine demonstrates several potential advantages when compared to alternative anesthetics. Midazolam is commonly employed as a first-line antiepileptic medication in RSE, but its use is limited by tachyphylaxis and diminishing efficacy over time [25]. Barbiturates can lead to rapid seizure suppression, but they are frequently associated with significant adverse effects, including hypotension, immunosuppression, and prolonged need for mechanical ventilation [26]. Propofol, despite its efficacy, carries the risk of propofol infusion syndrome, particularly during extended high-dose infusion, and it is also associated with hemodynamic instability [27]. In contrast, ketamine preserves cardiovascular stability, reduces excitotoxic neuronal injury through NMDA receptor antagonism, and is generally well tolerated [28,29].

Despite the lack of definitive trials, pooled observational data from our analysis suggest that ketamine demonstrates a comparable rate of seizure cessation to that reported for other second- or third-line therapies. However, this reflects an indirect comparison rather than evidence from controlled head-to-head studies.

This review has several strengths, including a comprehensive search strategy, inclusion of both adult and pediatric cohorts, and a pooled quantitative synthesis. Furthermore, it provides granular insight into dosing regimens, which varied widely across studies from loading boluses of 1-2 mg/kg to maintenance infusions ranging from 0.2 mg/kg/h up to 15 mg/kg/h. Despite this variability, seizure control was significantly improved, suggesting a broad therapeutic window.

Limitations must be acknowledged in this review. Most included studies were retrospective, with heterogeneous populations, variable or unclear underlying seizure etiologies, variable concomitant therapies, and variable outcome definitions. Many patients received multiple antiseizure medications before or during ketamine treatment, making it challenging to isolate ketamine’s independent effect. Small sample sizes in some cohorts, particularly pediatric series, limit generalizability. Additionally, adverse event reporting was inconsistent, and long-term neurodevelopmental outcomes were rarely addressed. Publication bias and selective outcome reporting cannot be excluded.

Future research should prioritize multicenter prospective registries and randomized controlled trials to establish standardized protocols for dosing, timing of initiation, and duration of therapy. Trials focusing on pediatric populations are especially warranted, given the encouraging but limited evidence base. Exploration of biomarkers of treatment response and incorporation of long-term functional outcomes will also be essential to fully define ketamine’s role in the management of RSE and SRSE.

## Conclusions

Intravenous ketamine shows potential efficacy in terminating seizures in RSE and SRSE, supporting its use as a therapeutic option when standard agents fail. However, the evidence is primarily derived from heterogeneous observational studies. With an overall seizure termination rate of 56% and comparable effectiveness between age groups, ketamine serves as a valuable therapeutic option when conventional GABAergic agents fail or are limited by hemodynamic instability. Its unique NMDA receptor antagonism, broad therapeutic window, and favorable safety profile further support its integration into treatment algorithms for severe SE. However, the evidence remains predominantly retrospective, with heterogeneous dosing strategies and limited pediatric data. Future prospective, standardized, and multicenter studies, particularly in children, are essential to clarify optimal dosing, timing of initiation, long-term outcomes, and safety, thereby refining ketamine’s role in the management of RSE and SRSE.
